# Limited neuropeptide Y precursor processing in unfavourable metastatic neuroblastoma tumours

**DOI:** 10.1054/bjoc.2000.1234

**Published:** 2000-06-15

**Authors:** P Bjellerup, E Theodorsson, H Jörnvall, P Kogner

**Affiliations:** Department of Clinical Chemistry, Department of Woman and Child Health, Karolinska Institutet and Karolinska Hospital, S-171 76 Stockholm; Childhood Cancer Research Unit, Department of Woman and Child Health, Karolinska Institutet and Karolinska Hospital, S-171 76 Stockholm; Department of Clinical Chemistry, University Hospital, S-581 85 Linköping; Department of Medical Biochemistry and Biophysics, Karolinska Institutet, S-171 77 Stockholm, Sweden

**Keywords:** neuropeptide Y, neuroblastoma, phaeochromocytoma, NPY processing, NPY (3–36), proNPY, prohormone processing

## Abstract

Neuropeptide Y (NPY) is found at high concentrations in neural crest-derived tumours and has been implicated as a regulatory peptide in tumour growth and differentiation. Neuroblastomas, ganglioneuromas and phaeochromocytomas with significant concentrations of NPY-like immunoreactivity were investigated for different molecular forms of NPY and for significance of proNPY processing. Gel-permeation chromatography identified intact NPY (1–36) in all tumours, whereas proNPY (69 amino acids) was detected only in control adrenal tissue and malignant neuroblastomas. Purification of NPY-like immunoreactivity in tumour extracts and structural characterization revealed that both NPY (1–36) and the truncated form NPY (3–36) was present. The degree of processing of proNPY to NPY in tumour tissue was lower in advanced neuroblastomas with regional or metastatic spread (stage 3 and 4) (*n* = 6), (41%, 12–100%, median, range), compared to the less aggressive stage 1, 2 and 4S tumours (*n* = 12), (93%; 69–100%), (*P* = 0.012). ProNPY processing of less than 50% was correlated with poor clinical outcome (*P* = 0.004). MYCN oncogene amplification was also correlated to a low degree of proNPY processing (*P* = 0.025). In summary, a low degree of proNPY processing was correlated to clinical advanced stage and poor outcome in neuroblastomas. ProNPY/NPY processing generated molecular forms of NPY with known differences in NPY-receptor selectivity, implicating a potential for in vivo modulation of NPY-like effects in tumour tissue. © 2000 Cancer Research Campaign


*British Journal of Cancer* (2000) **83**(2), 171–176
© 2000 Cancer Research Campaign
doi: 10.1054/ bjoc.2000.1234, available online at http://www.idealibrary.com on IDEAL®

### Limited neuropeptide Y precursor processing in unfavourable metastatic neuroblastoma tumours

**P Bjellerup[1], E Theodorsson[3], H Jörnvall[4] and P Kogner[2]**

[1]Department of Clinical Chemistry and [2]Childhood Cancer Research Unit, Department of Woman and Child Health, Karolinska Institutet and Karolinska Hospital, S-171 76 Stockholm; [3]Department of Clinical Chemistry, University Hospital, S-581 85 Linköping; [4]Department of Medical Biochemistry and Biophysics, Karolinska Institutet, S-171 77 Stockholm, Sweden

**Summary** Neuropeptide Y (NPY) is found at high concentrations in neural crest-derived tumours and has been implicated as a regulatory peptide in tumour growth and differentiation. Neuroblastomas, ganglioneuromas and phaeochromocytomas with significant concentrations of NPY-like immunoreactivity were investigated for different molecular forms of NPY and for significance of proNPY processing. Gel-permeation chromatography identified intact NPY (1–36) in all tumours, whereas proNPY (69 amino acids) was detected only in control adrenal tissue and malignant neuroblastomas. Purification of NPY-like immunoreactivity in tumour extracts and structural characterization revealed that both NPY (1–36) and the truncated form NPY (3–36) was present. The degree of processing of proNPY to NPY in tumour tissue was lower in advanced neuroblastomas with regional or metastatic spread (stage 3 and 4) ($n = 6$), (41%, 12–100%, median, range), compared to the less aggressive stage 1, 2 and 4S tumours ($n = 12$), (93%; 69–100%), ($P = 0.012$). ProNPY processing of less than 50% was correlated with poor clinical outcome ($P = 0.004$). MYCN oncogene amplification was also correlated to a low degree of proNPY processing ($P = 0.025$). In summary, a low degree of proNPY processing was correlated to clinical advanced stage and poor outcome in neuroblastomas. ProNPY/NPY processing generated molecular forms of NPY with known differences in NPY-receptor selectivity, implicating a potential for in vivo modulation of NPY-like effects in tumour tissue. © 2000 Cancer Research Campaign

**Key words**: neuropeptide Y; neuroblastoma; phaeochromocytoma; NPY processing; NPY (3–36); proNPY; prohormone processing

Neuroblastoma is the most common extra-cranial tumour in children. Its clinical behaviour ranges from spontaneous regression or complete remission after minimal drug therapy to unfavourable outcome from aggressive tumour growth despite intensive multimodal therapy (Katzenstein and Cohn, 1998). The benign differentiated counterpart, ganglioneuroma, usually has an excellent prognosis. Both tumours originate from the sympathetic nervous system and like all tumours of neuroendocrine origin they are able to produce different neuropeptides (Langley, 1994).

Measurement or identification of these neuropeptides has become increasingly important in screening, diagnosis and follow-up of several tumours or tumour syndromes (Pollak and Schally, 1998; Lee and Evans, 1997). Intervention by peptide analogues is a well established treatment in several modalities of neuroendocrine tumours and can relieve symptoms (Öberg, 1998).

Neuropeptide Y (NPY), a 36-residue peptide present throughout the peripheral and central nervous systems, is synthesized as a preprohormone, preproNPY (97 residues), first cleaved to proNPY (69 residues), and eventually to the biologically active, C-terminally amidated peptide, NPY, (1–36) (Lundberg et al, 1982; Adrian et al, 1983*a*; Allen, 1990). Both proNPY and NPY as well as other, not structurally identified, molecular forms of NPY-like immunoreactivity have been identified in neuroblastoma and pheochromocytoma tumours (Adrian et al, 1983*b*; O'Hare and Schwartz, 1989*b*; Kogner et al, 1993; deS Senanayake et al, 1995). Plasma concentrations, but not tumour concentrations, of NPY have been shown to be of value to monitor follow-up of treatment in neuroblastoma, and high concentrations have been associated with poor outcome (Kogner, 1995; Dötsch et al, 1998). Five different receptor subtypes for NPY have been cloned with different affinity profiles for NPY, truncated forms of NPY, and NPY-related peptides and several different physiological effects has been attributed to NPY through these receptors (Michel et al, 1998). NPY has also been implicated in cellular events, e.g. to be mitogenic in vascular smooth muscle cells and in neuroblastoma cells in vitro (Zukowska-Grojec et al, 1993; Shorter and Pence, 1997) and to be angiogenic both in vitro and in vivo, an effect mediated through the Y2-receptor (Zukowska-Grojec et al, 1998).

The aim of the present study was to investigate the processing of proNPY and its correlation to different clinical parameters. Analysis of proNPY processing was carried out using gel-permeation chromatography and an antiserum specific for the mid-portion of NPY. Peptides with NPY-like immunoreactivity (NPY-LI) were isolated, chromatographically purified and characterized by amino acid sequence analysis and mass spectrometry.

*Received 27 July 1999*
*Revised 6 January 2000*
*Accepted 13 March 2000*

*Correspondence to:* P Bjellerup

### MATERIAL AND METHODS

### Patient material and sample handling

Samples from primary tumours from 18 children with neuroblastoma and one child with ganglioneuroma containing significant

**Table 1** Results from measurements of NPY-LI and clinical data for individual patients.

| Pat. | Diagnosis | Age at diagnosis (months) | Gender | Follow-up (months) | Status | NPY-LI (pmol g$^{-1}$) | ProNPY (fmol) | NPY (fmol) | ProNPY-proc. (%) | MYCN ampl. (+/–) |
|---|---|---|---|---|---|---|---|---|---|---|
| T61 | AD | 41 | F | 88 | NED | 9.6 | 1487 | 806 | 35.1 | – |
| T41 | GN | 31 | F | 94 | NED | 2133 | 614 | 9570 | 93.8 | – |
| T63 | MET | 41 | F | 88 | NED | 545 | 2370 | 1979 | 45.5 | – |
| T90 | NB 1 | 13 | M | 61 | NED | 128 | 2162 | 4769 | 68.8 | – |
| T107 | NB 1 | 18 | F | 60 | NED | 682 | 2159 | 9755 | 81.9 | – |
| T29 | NB 2 | 0 | F | 118 | NED | 262 | 0 | 3392 | 100.0 | – |
| T92 | NB 2 | 12 | M | 62 | NED | 92 | 79 | 6204 | 98.7 | – |
| T116 | NB 2 | 33 | F | 52 | NED | 31.2 | 317 | 2692 | 89.5 | – |
| T65 | NB 2 | 103 | F | 73 | NED | 195 | 0 | 423 | 100.0 | – |
| T26 | NB 3 | 3 | F | 81 | NED | 451 | 2046 | 7904 | 79.4 | – |
| T64 | NB 3 | 6 | M | 80 | NED | 119 | 1248 | 676 | 35.1 | – |
| T93 | NB 3 | 21 | M | 6 | DOD | 203 | 1798 | 1644 | 47.8 | + |
| T38 | NB 4 | 8 | F | 4 | DOD | 282 | 2171 | 300 | 12.1 | + |
| T88 | NB 4 | 11 | F | 4 | DOD | 143 | 1437 | 483 | 25.2 | + |
| T50 | NB 4 | 24 | M | 10 | DOD | 226 | 6135 | 3149 | 33.9 | – |
| T52 | NB 4 | 32 | M | 18 | DOD | 116 | 634 | 6919 | 91.6 | – |
| T62 | NB 4 | 41 | F | 88 | NED | 78 | 0 | 328 | 100.0 | – |
| T51 | NB 4 | 60 | M | 48 | NED | 177 | 0 | 1289 | 100.0 | – |
| T01 | NB 4 | 60 | F | 38 | DOD | 220 | 1330 | 300 | 18.4 | – |
| T66 | NB 4S | 0 | M | 80 | NED | 50 | 580 | 3861 | 86.9 | – |
| T95 | NB 4S | 2 | F | 58 | NED | 55 | 32 | 901 | 96.6 | – |
| 8302 | PHEO | | | | | 1003 | 0 | 900 | 100.0 | |
| 8305 | PHEO | | | | | 259 | 0 | 1116 | 100.0 | |
| P7 | PHEO | | F | | | 305 | 293 | 3356 | 92.0 | |
| P8 | PHEO | | F | | | 3955 | 0 | 2122 | 100.0 | |
| P9 | PHEO | | M | | | 281 | 172 | 2842 | 94.3 | |

Healthy adrenal (AD), phaeochromocytomas (PHEO), ganglioneuromas (GN), neuroblastomas (NB) of different clinical stages (1–4 and 45) and metastasis (MET). No evidence of disease (NED), dead of disease (DOD)

concentrations of NPY-LI (30–4000 pmol g$^{-1}$), (Table 1) were selected from a series of tumours (Kogner, 1995). The tumours were diagnosed according to international criteria and neuroblastomas of all clinical stages were included (Brodeur et al, 1993). One metastasis, one healthy adrenal gland from a child with neuroblastoma, one ganglioneuroma and five phaeochromocytoma tumours from adult patients were also investigated. Tumour tissue, obtained at surgery, was frozen on solid $CO_2$ and kept at –70°C until extraction. Six of the children with neuroblastoma died within 4–38 months from diagnosis, whereas the remaining children have been followed for 48–118 months without any evidence of disease. The study was approved by the ethics committee of Karolinska Institutet, Stockholm, Sweden.

### Analytical peptide extraction

Extraction was performed by homogenization and boiling the tumour samples for 10 min in 10 volumes (minimum 2 ml) acetic acid (1 mol l$^{-1}$). After centrifugation at 2000 ***g*** for 15 min, the supernatants were lyophilized and dissolved in RIA-buffert.

### Preparative peptide extraction

Preparative extraction was performed on two samples from children with neuroblastoma. After homogenization, boiling, centrifugation and decantation, trifluoroacetic acid (TFA) was added to the supernatant to a final concentration of 0.1% and the material was loaded onto SepPak cartridges (Waters, Milford, MA, USA), primed with 10 ml 80% methanol, 0.1% TFA, and equilibrated with 10 ml water, 0.1% TFA. After loading the sample, the cartridge was flushed with 2 ml water, 0.1% TFA and immunoreactive material was eluted with 4 ml 80% methanol, 0.1% TFA. The eluates were dried under vacuum at 40°C, and dissolved in formic acid (1 mol l$^{-1}$), 0.02% sodium aside, before application onto gel-permeation chromatography.

### Gel-filtration and reversed-phase high-pressure liquid chromatography (RP-HPLC)

A Sephadex G-50 superfine column (2.6 × 95 cm) (Amersham Pharmacia Biotech, Sweden) was eluted with formic acid (1 mol l$^{-1}$), 0.02% sodium azide, 0.1% bovine serum albumin (BSA) (BSA was excluded in preparative runs) at a flow rate of 0.5 ml min$^{-1}$. The column was calibrated using synthetic NPY (1–36) and $^{3}$H-NPY (1–36) (Peninsula, Belmont, CA, USA). Void volume ($V_0$) was determined with Blue dextran and total volume ($V_t$) with $^{22}$Na. Fractions (2 ml in analytical and 8 ml in preparative runs) were collected and lyophilized for subsequent analysis for NPY-LI. For structural analysis, fractions were lyophilized and dissolved in 0.1% TFA, pooled and further purified by RP-HPLC.

RP-HPLC was carried out by C18 (4.6 × 250 mm) and C4 wide-pore (1 × 150 mm) columns (Vydac, Hesperia, CA, USA) using a conventional system and a SMART-system (Amersham Pharmacia Biotech), respectively. The columns were eluted (1 ml min$^{-1}$, and 30 µl min$^{-1}$, respectively) at room temperature with linear gradients of acetonitrile in water with 0.1% TFA or 0.1% TFA, 0.1% heptasulfonic acid. The eluate was monitored with UV-detection at 214 and 280 nm.

*© 2000 Cancer Research Campaign*

### Structural analysis

The primary structure of peptides was investigated by N-terminal amino acid sequence analysis using an Applied Biosystems 476 instrument (Perkin-Elmer, Norwalk, CT, USA) and phenyltiohydantion detection with a 120 analyser. Molecular mass were determined by MALDI-TOF mass spectrometry (Finnigan, San Jose, CA, USA). Both methods were carried out according to the manufacturers' instructions.

Fractions containing NPY immunoreactivity with a larger Stokes radius were lyophilized after gel-permeation chromatography and reconstituted in Veronal buffer, pH 8.6. Endoproteinase Lys-C (Sigma, St. Louis, MO, USA), 16 ng in 10 μl, was added prior to incubation at 37°C for 60 min.

### Radioimmunoassay (RIA)

NPY-LI was analysed with RIA performed under non-equilibrium conditions using intact porcine NPY (1–36) as standard. The antiserum was raised in rabbit and had specificity for the mid-portion of NPY (Theodorsson-Norheim et al, 1985). The cross-reactivity for human NPY (1–36) and (3–36) was 65.2 and 19.2%, respectively.

### MYCN oncogene amplification by Southern blot analysis

MYCN oncogene copy number was determined by Southern blot analysis performed as previously described (Hedborg et al, 1992) using the MYCN clone pNB 19–21. MYCN amplification was scored when the gene copy number was three or more in each haploid genome (Seeger et al, 1985).

### Calculation of processing ratio and statistics

The extent of proNPY-processing was calculated by dividing the amount of NPY-LI eluting at the position of NPY (1–36) with the amount of all NPY-LI. Significance was calculated according to Fischer's exact test, Mann–Whitney U test, Spearman and Pearson correlation coefficients. Survival probability was calculated according to Kaplan–Meier and compared using the logrank test.

### RESULTS

Gel-permeation chromatography identified two molecular forms of NPY-LI with different Stokes radii (Figure 1). Some of the neuroblastomas, the ganglioneuroma and the pheochromocytomas contained only little or nothing of the larger form of NPY-LI (first peak), (Table 1). Incubation with Endoproteinase Lys-C of the material with NPY-LI from the first peak (representing a larger Stokes radius) and subsequent gel-permeation chromatography, showed cleavage of the high molecular precursor to a component detected at a position slightly earlier than intact NPY (1–36) (data not shown). Incubation with 6 mol $l^{-1}$ urea for eliminating the possibility of protein binding or dimerization did not affect the result of gel-permeation chromatography (data not shown). The second peak, with a smaller Stokes radius, was present in all samples and eluted at the position of intact NPY (1–36)/$^3$H-NPY (1–36).

Tumour tissue from two children with neuroblastoma was further extracted on a preparative basis and gel-permeation chromatography was carried out. The second peak material with a smaller Stokes radius was further purified by RP-HPLC in several steps before amino acid sequence analysis. The result showed an amino acid sequence identical with that of human NPY (1–36) which was followed for 21 and 36 cycles, respectively. In the latter tumour, the amino-acid sequence analysis also revealed an N-terminally truncated form of NPY, NPY (3–36). This was confirmed by mass spectrometry, showing the molecular masses for the oxidized, C-terminally amidated forms of NPY (1–36) and NPY (3–36) (Figure 2).

The processing ratio in the healthy adrenal was 35% (Figure 3), (Table 1). In the ganglioneuroma and the adult pheochromocytomas it was more than 90% (98%, 92–100%, median, range), whereas the ratio in the neuroblastomas varied (80%, 12–100%). Metastatic tissue, available from one child, showed a processing ratio of 46%, as compared to 100% in the corresponding primary tumour. A significantly lower degree of proNPY processing was seen in neuroblastomas at advanced stage (stages 3 and 4) (41%, 12–100%) as compared to stages 1, 2 and 4S (93%, 69–100%, $P$ = 0.012) (Figure 3). A processing of less than 50% was significantly correlated with poor prognosis ($P$ = 0.004) and with a significant difference in survival probability ($P$ < 0.001, $\chi^2$ = 12.63) as analysed according to Kaplan–Meier (Figure 4). MYCN

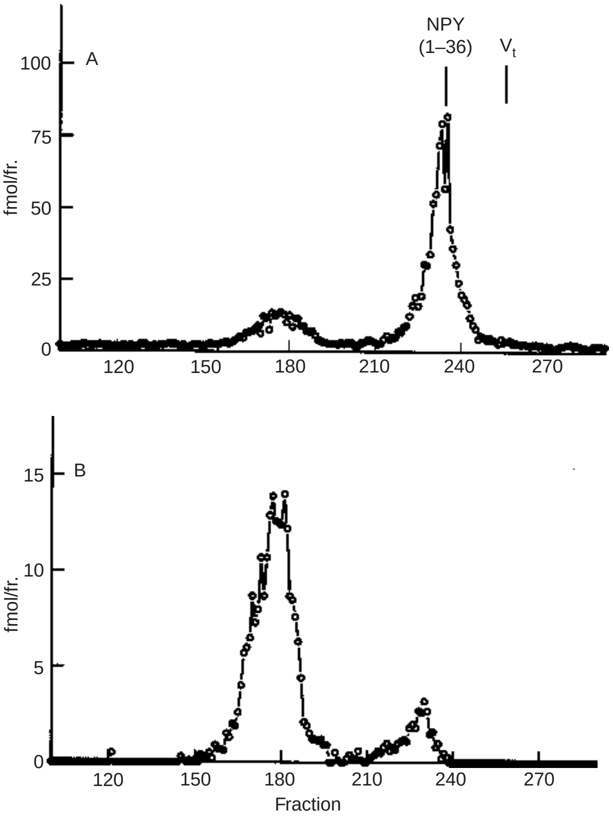

**Figure 1** Gel-permeation chromatography of NPY-LI in tumour tissue from a 3-month-old girl with neuroblastoma stage 3 (**A**) and an 8-month-old girl with MYCN amplified neuroblastoma stage 4 (**B**). The degree of proNPY processing was 74 and 12%. The girl with neuroblastoma stage 3 was alive and without evidence of disease after 81 months of follow-up, whereas the girl with neuroblastoma stage 4 died of disease after 8 months. The eluting position of intact NPY and total volume ($V_t$) is indicated. Void volume was at fraction number 75 (not shown).

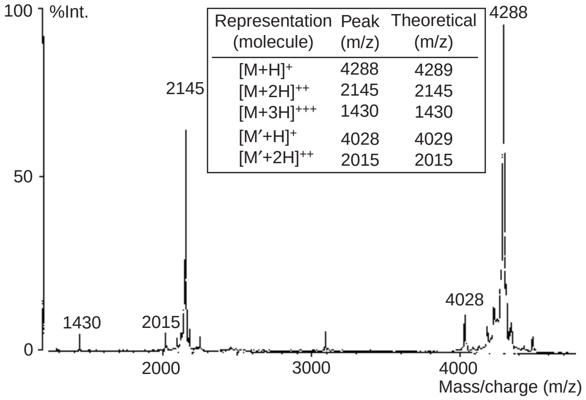

**Figure 2** Mass/charge chromatogram from MALDI-TOF mass spectrometry of NPY-LI from neuroblastoma tumour tissue. Material with NPY-LI was recovered from the gel-permeation chromatography eluting at the same position as intact NPY and subsequently purified in several steps with RP-HPLC. The molecular ions corresponding to sulfoxidized (methionine at position 17) intact NPY (1–36) (M) and N-terminally truncated form of NPY, NPY(3–36) (M′) with different charges (1+, 2+ and 3+) are indicated.

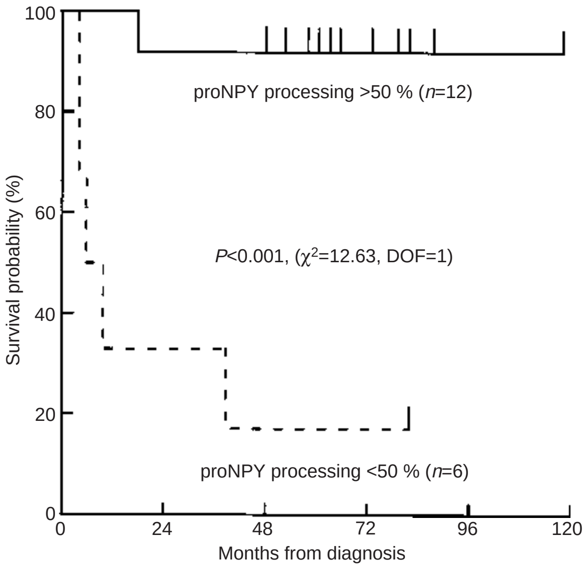

**Figure 4** Survival probability analysed according to Kaplan–Meier correlated to the degree of proNPY processing in 18 children with neuroblastoma. Survival probability for children with proNPY processing of more than 50% was 91.7 ± 8.0% ($n$ = 12) and for children with a processing of less than 50% it was 16.7 ± 15.2% ($n$ = 6) ($P$ < 0.001, $\chi^2$ = 12.63).

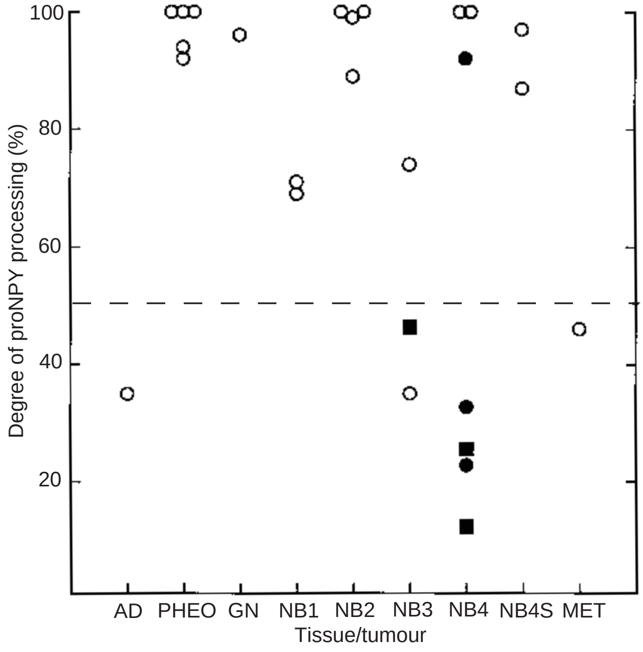

**Figure 3** Processing degree of proNPY to NPY (%) in adrenal and tumour tissue. AD, PHEO, GN, NB, and MET as in Table 1. Squares indicate MYCN amplification and filled symbols indicated children who died from the disease during follow-up. Broken line indicates 50% processing.

amplification was also significantly correlated to prognosis ($P$ = 0.025) and to a low degree of proNPY processing ($P$ = 0.025) but not to tumour stage ($P$ = 0.147) and there was a significant difference in survival probability as analysed according to Kaplan–Meier ($P$ < 0.001, $\chi^2$ = 21.19) (Figure 3). No other correlation was identified for the data in Table 1, e.g. there was no correlation between the concentration of NPY-LI and the processing degree using either Pearson ($r^2$ = 0.05, $P$ > 0.2) or Spearman ($P$ > 0.7) coefficients of variation.

### DISCUSSION

Pheochromocytomas and neuroblastomas may produce large amounts of NPY and the concentration of NPY in plasma can be a valuable marker in the diagnosis and monitoring of these diseases (Adrian et al, 1983*b*; Kogner et al, 1993; deS Senanayake et al, 1995). In the present material, a decreased intracellular processing of proNPY was significantly correlated with both widespread disease and poor outcome. Furthermore, a truncated form of NPY was identified indicating that the processing of proNPY and NPY in neuroblastoma may increase the possibility to modulate the interaction with different NPY receptors.

In this series of 25 tumours and one control adrenal, two major molecular forms of NPY-LI were identified (Figure 1). The larger molecular form of NPY-LI was deduced to be proNPY as was indicated by Endoproteinase Lys-C (Jekel et al, 1983). Endoproteinase Lys-C cleaves the peptide bond C-terminally of the Lys-residue, and can thereby be expected to cleave the 69 amino acid precursor proNPY C-terminally of the only lysine-residue present (position 38) in proNPY, generating the two fragments NPY (1–36)-Gly-Lys and Arg-CPON (C-flanking Peptide Of NPY) (O’Hare and Schwartz, 1989*a*).

The molecular form of NPY-LI with a smaller Stokes radius eluted at the same position as intact NPY. By subsequent purification and amino acid sequence analysis, intact NPY was identified in one tumour whereas the other tumour was shown to contain both intact NPY and an N-terminally truncated form of NPY, NPY (3–36), also confirmed with mass spectrometry (Figure 2).

The processing degree of proNPY to NPY in neuroblastoma was significantly higher in the more differentiated tumours as a processing of less than 50% was seen only in the most unfavourable tumours (stage 3 and 4) with regional or metastatic spread ($P$ = 0.012) (Figure 3). A processing of less than 50% was also significantly correlated with a poor survival probability as five of the six children in this group died within 38 months from

*© 2000 Cancer Research Campaign*

diagnosis (*P* = 0.004) (Figure 4). In the group of children with a processing of 50% or more, 11 out of 12 children were alive without evidence of disease during a follow-up time of 48 to 118 months.

Variation in the degree of processing is in agreement with an in vitro study using eight different neuroblastoma cell lines where the processing degree varied between 33 and 72% (O'Hare and Schwartz, 1989*a*). However, they are in contrast to another study by the same group where three neuroblastoma tumours all had more than 90% processing (O'Hare and Schwartz, 1989*b*). The difference in these results may be due to the fact that cell lines are most often raised from more malignant tumours, whereas the clinical tumours in the study may be of more benign stages (not specified in the articles).

Prohormone processing has been investigated in a number of endocrine tumours but has so far not been implicated to be of prognostic value (Rehfeld et al, 1996). The significant differences, although analysed in a limited number of samples, may indicate that biochemical events involved in the formation of NPY may be a part of an aggressive phenotype and that proNPY processing may be an indicator of prognosis in children with neuroblastoma.

The conversion of proNPY to the active peptide, NPY (1–36) occurs intracellulary within secretory vesicles involving several different enzymes. Among these enzymes the prohormone convertases (PC) 1 (also called PC 3) and PC 2 seem to be both the most substrate-specific and tissue-specific for proNPY processing (Wulff et al, 1993; Hook et al, 1996; Paquet et al, 1996). The present results may be derived from variation in PC-expression in the tumours; further studies are needed to elucidate this issue.

A low degree of proNPY processing in the more aggressive neuroblastomas may seem to contradict to the previous reported correlation between elevated concentrations of NPY-LI in plasma and poor outcome and relapse of disease (Kogner, 1995; Dötsch et al, 1998). However, the degree of proNPY processing is not correlated to the total amount of NPY-LI present (Table 1). Furthermore, no correlation between NPY mRNA expression and NPY-LI plasma concentration (tumour release) has been found (Dötsch et al, 1998). Thus, translation, transcription and cellular release are not necessarily linked.

The five pheochromocytomas from adult patients did not show significant amounts of proNPY. This is in agreement with previous studies of pheochromocytoma tumour extracts showing only one immunoreactive form of NPY (Adrian et al, 1983*b*; Corder et al, 1984; Allen et al, 1987; O'Hare and Schwartz, 1989*b*). No classification of the pheochromocytomas has been indicated in these previous studies, and also not in the present material. However, the amounts detected in all samples indicate that the processing degree in general, is high in pheochromocytoma tumour tissue.

Amplification of the MYCN oncogene is an established predictor of poor prognosis in neuroblastoma and is strongly correlated with clinically advanced stages (Seeger et al, 1985; Brodeur et al, 1992). In the present study, all three tumours with MYCN amplification showed a low degree of proNPY processing, were of advanced clinical stage and the children died during follow-up (Table 1 and Figure 3). However, a low degree of proNPY-processing had a higher correlation to advanced tumour stage and poor outcome than MYCN amplification.

By amino acid sequence analysis and mass spectrometry, intact NPY was identified in one tumour investigated, whereas another tumour was shown to contain both intact NPY and an N-terminally truncated form of NPY, NPY (3–36) (Figure 2). Evidently, none of the chromatographic methods, or the RIA, was able to separate intact NPY from NPY (3–36). This lack of specificity is probably a feature that is in common with most, if not all, other investigations.

NPY (3–36) has earlier been identified in a human somatostatinoma of the pancreas but has, to our knowledge, not been found in normal human tissue or blood (Shaw et al, 1993). NPY has an N-terminally tyrosine followed by a prolyl residue. This structure renders the peptide resistant to generalized aminopeptidase degradation but susceptible to more specialized enzymes (Mentlein, 1988). Several enzymes have recently been investigated and human dipeptidyl amino peptidase (DPP) IV was the only enzyme with high activity for NPY generating Tyr-Pro dipeptides (Mentlein et al, 1993). DPP IV has been identified on the surface of endothelial cells, T-lymphocytes (CD 26), hepatocytes and in the intestinal and kidney brush border membranes, but adrenal or tumour tissue has not been investigated (Mentlein et al, 1993).

The multitude of receptors and ligands indicate that the processing of proNPY and intact NPY offers a potential to modulate the physiological and biological actions of NPY (Michel et al, 1998). Processing of NPY to NPY (3–36) generates a molecule, which does not bind to the Y1 receptor (Grandt et al, 1996). The presence of both intact NPY and NPY (3–36) in the same tumour tissue has not been reported earlier. These findings indicate the presence of DPP IV on the surface of cells in neuroblastoma tumours, converting non-receptor selective intact NPY into Y2-, Y3- and Y5 receptor-selective NPY (3–36), thereby creating a potential for modulation, or even a shift, in biological actions. The Y1 and Y2 receptor may be expressed in neuroblastoma cell lines. (Wahlestedt et al, 1992). and formation of NPY (3–36) in neuroblastoma tumours may be of particular significance since the Y2 receptor mediates the angiogenic activity of the ligand NPY (3–36) in vitro and in vivo (Zukowska-Grojec et al, 1998).

In conclusion, a low degree of proNPY processing in tumour tissue was correlated to advanced disease with regional or metastatic spread and poor outcome in neuroblastoma. ProNPY/NPY processing within the same tumour tissue generated NPY (3–36), a molecule known to have different receptor selectivity in comparison with intact NPY. Simple methods are needed to specifically measure the different forms of NPY-LI that are present in neuroblastoma tumour tissue.

### ACKNOWLEDGEMENTS

This work was supported by The Children's Cancer Foundation of Sweden, The Cancer Research Fund (1806, 2313, and 3165) and The Swedish Society for Medical Research.

### REFERENCES

Adrian TE, Allen JM, Bloom SR, Ghatei MA, Rossor MN, Roberts GW, Crow TJ, Tatemoto K and Polak JM (1983*a*) Neuropeptide Y distribution in human brain. *Nature* **306**: 584–586

Adrian TE, Allen JM, Terenghi G, Bacarese-Hamilton AJ, Brown MJ, Polak JM and Bloom SR (1983*b*) Neuropeptide Y in phaeochromocytomas and ganglioneuroblastomas. *Lancet* **2**: 540–542

Allen JM (1990) Molecular structure of neuropeptide Y. *Ann N Y Acad Sci* **611**: 86–98

Allen JM, Yeats JC, Causon R, Brown MJ and Bloom SR (1987) Neuropeptide Y and its flanking peptide in human endocrine tumors and plasma. *J Clin Endocrinol Metab* **64**: 1199–1204

Brodeur GM, Azar C, Brother M, Hiemstra J, Kaufman B, Marshall H, Moley J, Nakagawara A, Saylors R, Scavarda N, Schneider ES, Wasson J, White P, Seeger R, Look T and Castleberry R (1992) Neuroblastoma. Effect of genetic factors on prognosis and treatment. *Cancer* **70**: 1685–1694

Brodeur GM, Pritchard J, Berthold F, Carlsen NL, Castel V, Castelberry RP, De Bernardi B, Evans AE, Favrot M, Hedborg F, Kaneko M, Kemshead J, Lampert F, Lee REJ, Look AT, Pearson ADJ, Philip T, Roald B, Sawada T, Seeger RC, Tsuchida Y and Voute PA (1993) Revisions of the international criteria for neuroblastoma diagnosis, staging, and response to treatment [see comments]. *J Clin Oncol* **11**: 1466–1477

Corder R, Emson PC and Lowry PJ (1984) Purification and characterization of human neuropeptide Y from adrenal-medullary phaeochromocytoma tissue. *Biochem J* **219**: 699–706

deS Senanayake P, Denker J, Bravo EL and Graham RM (1995) Production, characterization, and expression of neuropeptide Y by human pheochromocytoma. *J Clin Invest* **96**: 2503–2509

Dötsch J, Christiansen H, Hanze J, Lampert F and Rascher W (1998) Plasma neuropeptide Y of children with neuroblastoma in relation to stage, age and prognosis, and tissue neuropeptide Y. *Regul Pept* **75–76**: 185–190

Grandt D, Schimiczek M, Rascher W, Feth F, Shively J, Lee TD, Davis MT, Reeve JR, Jr and Michel MC (1996) Neuropeptide Y 3–36 is an endogenous ligand selective for Y2 receptors. *Regul Pept* **67**: 33–37

Hedborg F, Lindgren PG, Johansson I, Kogner P, Samuelsson BO, Bekassy AN, Olsen L, Kreuger A and Påhlman S (1992) N-myc gene amplification in neuroblastoma: a clinical approach using ultrasound guided cutting needle biopsies collected at diagnosis. *Med Pediatr Oncol* **20**: 292–300

Hook VY, Schiller MR and Azaryan AV (1996) The processing proteases prohormone thiol protease, PC1/3 and PC2, and 70-kDa aspartic proteinase show preferences among proenkephalin, proneuropeptide Y, and proopiomelanocortin substrates. *Arch Biochem Biophys* **328**: 107–114

Jekel PA, Weijer WJ and Beintema JJ (1983) Use of endoproteinase Lys-C from Lysobacter enzymogenes in protein sequence analysis. *Anal Biochem* **134**: 347–354

Katzenstein HM and Cohn SL (1998) Advances in the diagnosis and treatment of neuroblastoma. *Curr Opin Oncol* **10**: 43–51

Kogner P (1995) Neuropeptides in neuroblastomas and ganglioneuromas. *Prog Brain Res* **104**: 325–338

Kogner P, Björk O and Theodorsson E (1993) Neuropeptide Y in neuroblastoma: increased concentration in metastasis, release during surgery, and characterization of plasma and tumor extracts. *Med Pediat Oncol* **21**: 317–322

Langley K (1994) The neuroendocrine concept today. *Ann N Y Acad Sci* **733**: 1–17

Lee JE and Evans DB (1997) Advances in the diagnosis and treatment of gastrointestinal neuroendocrine tumors. *Cancer Treat Res* **90**: 227–238

Lundberg JM, Terenius L, Hökfelt T, Martling CR, Tatemoto K, Mutt V, Polak J, Bloom S and Goldstein M (1982) Neuropeptide Y (NPY)-like immunoreactivity in peripheral noradrenergic neurons and effects of NPY on sympathetic function. *Acta Physiol Scand* **116**: 477–480

Mentlein R (1988) Proline residues in the maturation and degradation of peptide hormones and neuropeptides. *FEBS Lett* **234**: 251–256

Mentlein R, Dahms P, Grandt D and Kruger R (1993) Proteolytic processing of neuropeptide Y and peptide YY by dipeptidyl peptidase IV. *Regul Pept* **49**: 133–144

Michel MC, Beck-Sickinger A, Cox H, Doods HN, Herzog H, Larhammar D, Quirion R, Schwartz T and Westfall T (1998) XVI. International Union of Pharmacology recommendations for the nomenclature of neuropeptide Y, peptide YY, and pancreatic polypeptide receptors. *Pharmacol Rev* **50**: 143–150

O’Hare MM and Schwartz TW (1989*a*) Expression and precursor processing of neuropeptide Y in human and murine neuroblastoma and pheochromocytoma cell lines. *Cancer Res* **49**: 7015–7019

O’Hare MM and Schwartz TW (1989*b*) Expression and precursor processing of neuropeptide Y in human pheochromocytoma and neuroblastoma tumors. *Cancer Res* **49**: 7010–7014

Öberg K (1998) Advances in chemotherapy and biotherapy of endocrine tumors. *Curr Opin Oncol* **10**: 58–65

Paquet L, Massie B and Mains RE (1996) Proneuropeptide Y processing in large dense-core vesicles: manipulation of prohormone convertase expression in sympathetic neurons using adenoviruses. *J Neurosci* **16**: 964–973

Pollak MN and Schally AV (1998) Mechanisms of antineoplastic action of somatostatin analogs. *Proc Soc Exp Biol Med* **217**: 143–152

Rehfeld JF, Bardram L and Hilsted L (1996) Gastroenteropancreatic tumours and prohormones. *Scand J Gastroenterol Suppl* **216**: 39–45

Seeger RC, Brodeur GM, Sather H, Dalton A, Siegel SE, Wong KY and Hammond D (1985) Association of multiple copies of the N-myc oncogene with rapid progression of neuroblastomas. *N Engl J Med* **313**: 1111–1116

Shaw C, Cormican K, Thim L, Maule AG, Sloan JM and Buchanan KD (1993) Neuropeptide Y and neuropeptide Y 3–36: isolation from human pancreatic endocrine tumours. *Regul Pept* **45**: 387–394

Shorter NA and Pence JC (1997) Retinoic acid-induced regulation of neuropeptide Y receptor expression and function in the neuroepithelioma line SK-N-MC. *J Pediatr S* **32**: 721–723

Theodorsson-Norheim E, Hemsen A and Lundberg JM (1985) Radioimmunoassay for neuropeptide Y (NPY): chromatographic characterization of immunoreactivity in plasma and tissue extracts. *Scand J Clin Lab Invest* **45**: 355–365

Wahlestedt C, Regunathan S and Reis DJ (1992) Identification of cultured cells selectively expressing Y1-, Y2-, or Y3-type receptors for neuropeptide Y/peptide YY. *Life Sci* **50**: PL7–12

Wulff BS, Johansen TE, Dalboge H, O’Hare MM and Schwartz TW (1993) Processing of two homologous precursors, pro-neuropeptide Y and pro-pancreatic polypeptide, in transfected cell lines expressing different precursor convertases. *J Biol Chem* **268**: 13327–13335

Zukowska-Grojec Z, Karwatowska-Prokopczuk E, Rose W, Rone J, Movafagh S, Ji H, Yeh YY, Chen WT, Kleinman HK, Grouzmann E and Grant DS (1998) Neuropeptide Y – A novel angiogenic factor from the sympathetic nerves and endothelium. *Circ Res* **83**: 187–195

Zukowska-Grojec Z, Pruszczyk P, Colton C, Yao J, Shen GH, Myers AK and Wahlestedt C (1993) Mitogenic effect of neuropeptide Y in rat vascular smooth muscle cells. *Peptides* **14**: 263–268

 *© 2000 Cancer Research Campaign*

## References

[PDF_00514] Adrian T. E., Allen J. M., Bloom S. R., Ghatei M. A., Rossor M. N., Roberts G. W., Crow T. J., Tatemoto K., Polak J. M. (1983). Neuropeptide Y distribution in human brain.. Nature.

[PDF_00517] Adrian T. E., Allen J. M., Terenghi G., Bacarese-Hamilton A. J., Brown M. J., Polak J. M., Bloom S. R. (1983). Neuropeptide Y in phaeochromocytomas and ganglioneuroblastomas.. Lancet.

[PDF_00520] Allen J. M. (1990). Molecular structure of neuropeptide Y.. Ann N Y Acad Sci.

[PDF_00522] Allen J. M., Yeats J. C., Causon R., Brown M. J., Bloom S. R. (1987). Neuropeptide Y and its flanking peptide in human endocrine tumors and plasma.. J Clin Endocrinol Metab.

[PDF_00527] Brodeur G. M., Azar C., Brother M., Hiemstra J., Kaufman B., Marshall H., Moley J., Nakagawara A., Saylors R., Scavarda N. (1992). Neuroblastoma. Effect of genetic factors on prognosis and treatment.. Cancer.

[PDF_00531] Brodeur G. M., Pritchard J., Berthold F., Carlsen N. L., Castel V., Castelberry R. P., De Bernardi B., Evans A. E., Favrot M., Hedborg F. (1993). Revisions of the international criteria for neuroblastoma diagnosis, staging, and response to treatment.. J Clin Oncol.

[PDF_00537] Corder R., Emson P. C., Lowry P. J. (1984). Purification and characterization of human neuropeptide Y from adrenal-medullary phaeochromocytoma tissue.. Biochem J.

[PDF_00543] Dötsch J., Christiansen H., Hänze J., Lampert F., Rascher W. (1998). Plasma neuropeptide Y of children with neuroblastoma in relation to stage, age and prognosis, and tissue neuropeptide Y.. Regul Pept.

[PDF_00546] Grandt D., Schimiczek M., Rascher W., Feth F., Shively J., Lee T. D., Davis M. T., Reeve J. R., Michel M. C. (1996). Neuropeptide Y 3-36 is an endogenous ligand selective for Y2 receptors.. Regul Pept.

[PDF_00549] Hedborg F., Lindgren P. G., Johansson I., Kogner P., Samuelsson B. O., Bekassy A. N., Olsen L., Kreuger A., Påhlman S. (1992). N-myc gene amplification in neuroblastoma: a clinical approach using ultrasound guided cutting needle biopsies collected at diagnosis.. Med Pediatr Oncol.

[PDF_00553] Hook V. Y., Schiller M. R., Azaryan A. V. (1996). The processing proteases prohormone thiol protease, PC1/3 and PC2, and 70-kDa aspartic proteinase show preferences among proenkephalin, proneuropeptide Y, and proopiomelanocortin substrates.. Arch Biochem Biophys.

[PDF_00557] Jekel P. A., Weijer W. J., Beintema J. J. (1983). Use of endoproteinase Lys-C from Lysobacter enzymogenes in protein sequence analysis.. Anal Biochem.

[PDF_00560] Katzenstein H. M., Cohn S. L. (1998). Advances in the diagnosis and treatment of neuroblastoma.. Curr Opin Oncol.

[PDF_00564] Kogner P., Björk O., Theodorsson E. (1993). Neuropeptide Y in neuroblastoma: increased concentration in metastasis, release during surgery, and characterization of plasma and tumor extracts.. Med Pediatr Oncol.

[PDF_00562] Kogner P. (1995). Neuropeptides in neuroblastomas and ganglioneuromas.. Prog Brain Res.

[PDF_00568] Langley K. (1994). The neuroendocrine concept today.. Ann N Y Acad Sci.

[PDF_00570] Lee J. E., Evans D. B. (1997). Advances in the diagnosis and treatment of gastrointestinal neuroendocrine tumors.. Cancer Treat Res.

[PDF_00573] Lundberg J. M., Terenius L., Hökfelt T., Martling C. R., Tatemoto K., Mutt V., Polak J., Bloom S., Goldstein M. (1982). Neuropeptide Y (NPY)-like immunoreactivity in peripheral noradrenergic neurons and effects of NPY on sympathetic function.. Acta Physiol Scand.

[PDF_00579] Mentlein R., Dahms P., Grandt D., Krüger R. (1993). Proteolytic processing of neuropeptide Y and peptide YY by dipeptidyl peptidase IV.. Regul Pept.

[PDF_00577] Mentlein R. (1988). Proline residues in the maturation and degradation of peptide hormones and neuropeptides.. FEBS Lett.

[PDF_00582] Michel M. C., Beck-Sickinger A., Cox H., Doods H. N., Herzog H., Larhammar D., Quirion R., Schwartz T., Westfall T. (1998). XVI. International Union of Pharmacology recommendations for the nomenclature of neuropeptide Y, peptide YY, and pancreatic polypeptide receptors.. Pharmacol Rev.

[PDF_00587] O'Hare M. M., Schwartz T. W. (1989). Expression and precursor processing of neuropeptide Y in human and murine neuroblastoma and pheochromocytoma cell lines.. Cancer Res.

[PDF_00590] O'Hare M. M., Schwartz T. W. (1989). Expression and precursor processing of neuropeptide Y in human pheochromocytoma and neuroblastoma tumors.. Cancer Res.

[PDF_00593] Oberg K. (1998). Advances in chemotherapy and biotherapy of endocrine tumors.. Curr Opin Oncol.

[PDF_00595] Paquet L., Massie B., Mains R. E. (1996). Proneuropeptide Y processing in large dense-core vesicles: manipulation of prohormone convertase expression in sympathetic neurons using adenoviruses.. J Neurosci.

[PDF_00599] Pollak M. N., Schally A. V. (1998). Mechanisms of antineoplastic action of somatostatin analogs.. Proc Soc Exp Biol Med.

[PDF_00601] Rehfeld J. F., Bardram L., Hilsted L. (1996). Gastroenteropancreatic tumours and prohormones.. Scand J Gastroenterol Suppl.

[PDF_00603] Seeger R. C., Brodeur G. M., Sather H., Dalton A., Siegel S. E., Wong K. Y., Hammond D. (1985). Association of multiple copies of the N-myc oncogene with rapid progression of neuroblastomas.. N Engl J Med.

[PDF_00607] Shaw C., Cormican K., Thim L., Maule A. G., Sloan J. M., Buchanan K. D. (1993). Neuropeptide Y and neuropeptide Y 3-36: isolation from human pancreatic endocrine tumours.. Regul Pept.

[PDF_00610] Shorter N. A., Pence J. C. (1997). Retinoic acid-induced regulation of neuropeptide Y receptor expression and function in the neuroepithelioma line SK-N-MC.. J Pediatr Surg.

[PDF_00613] Theodorsson-Norheim E., Hemsén A., Lundberg J. M. (1985). Radioimmunoassay for neuropeptide Y (NPY): chromatographic characterization of immunoreactivity in plasma and tissue extracts.. Scand J Clin Lab Invest.

[PDF_00620] Wulff B. S., Johansen T. E., Dalbøge H., O'Hare M. M., Schwartz T. W. (1993). Processing of two homologous precursors, pro-neuropeptide Y and pro-pancreatic polypeptide, in transfected cell lines expressing different precursor convertases.. J Biol Chem.

[PDF_00624] Zukowska-Grojec Z., Karwatowska-Prokopczuk E., Rose W., Rone J., Movafagh S., Ji H., Yeh Y., Chen W. T., Kleinman H. K., Grouzmann E. (1998). Neuropeptide Y: a novel angiogenic factor from the sympathetic nerves and endothelium.. Circ Res.

[PDF_00628] Zukowska-Grojec Z., Pruszczyk P., Colton C., Yao J., Shen G. H., Myers A. K., Wahlestedt C. (1993). Mitogenic effect of neuropeptide Y in rat vascular smooth muscle cells.. Peptides.

[PDF_00540] deS Senanayake P., Denker J., Bravo E. L., Graham R. M. (1995). Production, characterization, and expression of neuropeptide Y by human pheochromocytoma.. J Clin Invest.

